# Comparison of Mechanical and Crack-Healing Properties of PE-PVA Hybrid Fiber-Reinforced SHCCs in Natural and Underwater Conditions

**DOI:** 10.3390/ma15186339

**Published:** 2022-09-13

**Authors:** Se-Eon Park, Huy Hoàng Nguyễn, Jeong-Il Choi, Bang Yeon Lee, Yun Yong Kim

**Affiliations:** 1 Department of Architecture and Civil Engineering, Chonnam National University, Gwangju 61186, Korea; 2 Biohousing Research Center, Chonnam National University, Gwangju 61186, Korea; 3 School of Architecture, Chonnam National University, Gwangju 61186, Korea; 4 Department of Civil Engineering, Chungnam National University, 99 Daehak-ro, Yuseong-gu, Daejeon 34134, Korea

**Keywords:** strain hardening cementitious composite, mechanical properties, crack-healing, fiber hybridization

## Abstract

This paper presents a direct comparison of the mechanical and crack-healing properties of strain hardening cementitious composites (SHCC) under water submersion in a laboratory and in a natural environment outdoors. Portland cement, slag, crumb rubber powder, and hybrid polyethylene and polyvinyl alcohol fibers were used for the SHCC, and mixture proportions were determined. Specimens were exposed to different environmental conditions. A sequence of experimental tests including those for density, compressive strength, and tensile properties was performed to assess the mechanical properties of the SHCC. To confirm the healing feasibility of the SHCC, crack width reduction, stiffness recovery, and tensile performance at post-healing were adopted. The test results showed that underwater conditions are better than natural conditions in improving both the mechanical and crack-healing properties of SHCC. Specifically, the SHCC cured in natural conditions had a lower compressive strength, tensile strength, and tensile strain capacity than that cured in underwater conditions by 10%, 4%, and 3%, respectively. The SHCC cured in underwater conditions had a healing threshold of crack width of 60 µm, while the SHCC cured in natural conditions had very limited crack-healing capacity. Additionally, stiffness recovery of the SHCC cured in underwater conditions was higher than that cured in natural conditions.

**Highlights**

- Effects of environmental conditions on properties of SHCC were investigated.
- SHCC exposed to natural environment showed slightly lower mechanical properties.
- SHCC exposed to natural environment showed slight crack-sealing performance.
- Both SHCCs under different conditions showed similar trends in tensile behavior at reloading.

## 1. Introduction

Conventional concrete is the most used construction material worldwide [1]. However, concrete is prone to cracking at an early age or after long-term use, making it a risky material for construction engineering [2]. Cracking may reduce the structural performance and durability of concrete members; therefore, costly maintenance and repair are required. Another drawback of concrete is that the manufacture of Portland cement, the main ingredient of concrete, releases large amounts of carbon dioxide and requires high energy consumption [3,4].

Highly ductile fiber-reinforced concrete, or strain hardening cementitious composite (SHCC), was developed by Li et al. (1990) to overcome the inherent brittleness of normal concrete [5]. SHCC was designed based on fracture mechanics and steady-state cracking theory: matrix, fiber, and interfacial properties between matrix and fiber were tailored for strain-hardening and multiple cracking behavior of composites. Although SHCC is a subset of fiber-reinforced concrete (FRC), its tensile ductility is several hundred times higher than that of FRC. Unlike FRC under tension, SHCC shows multiple cracks with tiny crack width, commonly below 100 µm. Interestingly, cracks of the SHCC members can be sealed automatically without either internal or external support, called “crack-healing”; the crack-healing mechanism involves the further hydration of Portland cement under water excitation and bridging fibers that attract healing materials to fill crack gaps. Because of the small crack width and the presence of fibers across cracks, SHCC has a higher crack-healing capacity than those of FRC or normal concrete, which may result in improvements in resilience and durability, as well as the low maintenance cost of members or structures made of SHCC [6].

Yang et al. were the first to investigate the crack-healing properties of SHCC reinforced by polyvinyl alcohol (PVA) fiber under wet-dry cycles [6,7]. Their results indicate that crack width smaller than 50 µm led to full recovery; crack width from 50 µm to 150 µm showed partial recovery; and crack width larger than 150 µm resulted in no healing potential. Later, there were many investigations into the effects of ingredients, curing methods, healing agents, etc., on the crack-healing behavior of SHCC using PVA fiber as main reinforcement [8,9,10,11,12]. PVA fiber, with its hydrophilic surface, facilitates precipitation of healing materials within micro-cracks [13]. Recently, because of its excellent physical properties, polyethylene (PE) fiber has been adopted to replace PVA fiber in SHCC in order to not only enhance tensile performance but also retain good healing properties [14,15,16,17,18]. However, the hydrophobic nature of the PE fiber means that it cannot be used to obtain greater healing capacity than that possible using PVA fiber. Furthermore, PE fiber is about five times more expensive than PVA fiber [19].

Incorporating both PVA and PE fibers can thus be a new method of developing high-ductile SHCCs with crack-healing ability; PVA fiber enables crack-healing potential, whereas PE fiber enhances tensile ductility. Studies of hybrid fiber-reinforced SHCCs with metallic/alloy and polymeric fibers, i.e., steel-PVA, shape memory alloy-PVA, and steel-PE, have been reported [20,21,22,23]; however, studies of polymeric fiber hybridization, i.e., PVA-PE, have been fairly limited [24]. Furthermore, it is necessary to investigate the mechanical and crack-healing properties of SHCC under in situ environmental exposure. Although a few studies have investigated the crack-healing properties of SHCCs exposed to natural environments [2,25], the literature on direct comparisons between properties of SHCCs in natural outdoor environments and under water submersion in the laboratory is fairly limited. This study addresses knowledge gaps and seeks previously unavailable information on SHCCs, investigating the effects of the natural environment on the composite properties, i.e., density, compressive strength, tensile performance, and crack-healing of hybrid fiber-reinforced SHCCs, comparing these properties with those obtained under water-submersed conditions. Crack-healing properties of SHCCs were evaluated in terms of degree of crack closure, stiffness recovery, and tensile behavior at reloading in post-healing phase.

## 2. Experimental Program

### 2.1. Materials and Mixture Proportions

Type I Portland cement and ground granulated blast furnace slag (GGBS) were the SHCC binding materials. High volume GGBS was used to increase the sustainability of SHCC. The chemical components of the cement and GGBS, determined by X-ray fluorescence (PANalytical), are shown in Table 1. Crumb rubber (CR) powder (size of 40 mesh) and silica sand (SS) were used as fine aggregate. CR is made from recycled worn-out car tires; it was used to increase the tensile strain capacity as well as the sustainability of SHCC [26]. Expansive agent (EA) was adopted to reduce chemical and drying shrinkages of SHCC. Furthermore, optimal dosages of superplasticizer (SP) and viscosity modifying admixture (VMA) were used to achieve proper rheological properties of matrix for good fiber distribution. A defoamer (DF) was added to prevent the formation of bubbles during the mixing process. Short hybrid PE and PVA micro-fibers were used as reinforcement. The physical properties of the PE and PVA fibers are listed in Table 2.

Table 3 provides details of mixture proportions, based on total binder weight. There was one mix design, but it was subjected to two dissimilar curing regimes. M-W indicates the mixture cured in underwater condition (U-C), whereas M-O denotes the mixture cured in natural condition (N-C). GGBS was used to replace 50 wt% of Type I Portland cement in SHCC. Water-to-binder ratio (*w*/*b*) was 0.4 and sum of fiber volume fraction was 1.35, consisting of 1.25% and 0.10% volumetric amounts of PE and PVA fiber, respectively.

### 2.2. Specimen Preparation

The raw materials, including binders, EA, CR powder, and SS, were initially dry-mixed for three minutes at 90 revolutions per minute (rpm). Then, water, SP, VMA, and DF were simultaneously added to the dry mixture and mixed for another three minutes at 270 rpm. Once the fresh mixture had become homogenous, PE and PVA fibers were slowly inserted into the mixture at 90 rpm; the mixture was later mixed at 270 rpm until the fibers were evenly distributed. No fiber agglomeration or segregation were found after the mixing process. The fresh SHCC was cast into a 50 mm cube and dumbbell-shape molds for density/compression and tensile/crack-healing tests, respectively. A total of three cube specimens and eight tensile specimens were prepared for both MO and MW mixtures. Three out of eight tensile specimens were used to evaluate the crack-healing properties.

All specimens were cured at room temperature (23 °C ± 3 °C) for 2 days; they were then de-molded and stored in specific curing regimes (U-C and N-C) until 28 days of age. In detail, the M-W mixture was subjected to U-C at room temperature, while the M-O mixture was exposed to N-C. All experiments took place at Chonnam National University (Gwangju, Republic of Korea). At 28 days of age, specimens arranged for crack-healing evaluation were preloaded to create initial cracks and later subjected to continued curing under the previous two sets of conditions for a further 28 days. Table 4 shows information on the natural environment that M-O mixture was subjected to.

### 2.3. Density and Mechanical Measurements

Density of SHCC was measured using cube specimens at 28 days of age, based on their weights in air ($m_{a}$) and water ($m_{b}$). Equation (1) shows the equation used to calculate the density (ρ).

(1) $$\rho =\frac{m_{a}}{m_{a}-m_{b}}\times \rho_{w}$$

where $\rho_{w}$ is water density, 1 g/cm^3^.

**Figure 1:**
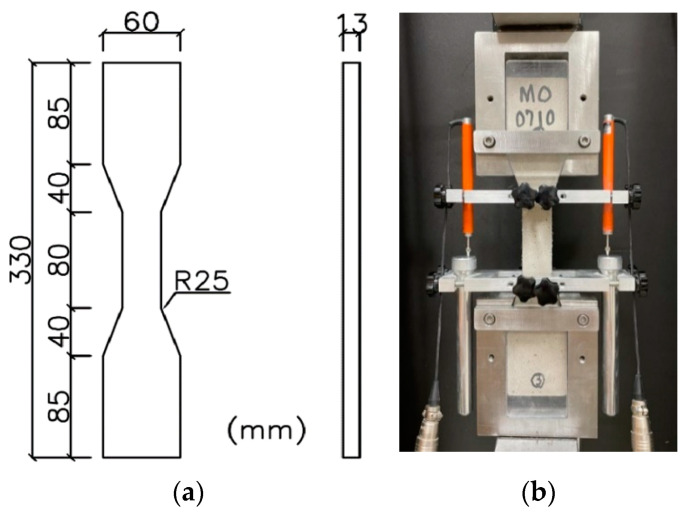
(**a**) Specimen geometry and (**b**) tension test setup.

At 28 days of age, a compressive strength test was performed in accordance with ASTM C109/109M using a compression test machine with a load capacity up to 300 kN [27]. Using a tension machine with a maximum load capacity of 20 kN, tension tests were executed at 28 days of age according to recommendations of the Japan Society of Civil Engineering [28] for high performance fiber reinforced composites. Figure 1 shows the geometry of tensile specimens and setup of tension tests. A load cell was fixed at the top of the machine to measure the tensile load applied to the specimens; two linear variable differential transducers were mounted vertically on both sides of the specimens to record the tensile deformation over a gauge length of 80 mm. Cracking patterns in terms of the number of cracks, crack width, and crack spacing were quantified after the tension test.

### 2.4. Crack-Healing Assessment

As has been reported, a decrease in crack width ($W_{c}$) is a recognizable signal of crack-healing concrete. Therefore, crack width reduction was adopted to assess the crack-healing capacity of SHCC. Tensile specimens were pre-cracked by deforming them under uniaxial tension at 3% strain at 28 days of age. $W_{c}$ values of specimens were initially recorded using a digital microscope (Sometech, Seoul, Korea) at 84× magnification. After a further 28 days, residual $W_{c}$ values of specimens were recorded again and compared to those at pre-loading to evaluate the crack-healing performance.

The resonant frequency (RF)-based non-destructive technique has been widely used to assess the crack-healing and stiffness recovery of SHCC [29]. In detail, crack-healing occurs if RF at post-healing is higher than that at pre-cracking. The stiffness recovery can be evaluated through RF restoration from pre-cracking to post-healing phases. The RF test configuration and setup are described in the literature [14,15]. After 28 healing days (56 days of age), the healed tensile specimens were re-loaded until failure and their crack widths were compared to those at 28 days of age to confirm the feasibility of tensile restoration of SHCC.

## 3. Results and Discussion

### 3.1. Mechanical Properties

**Figure 2:**
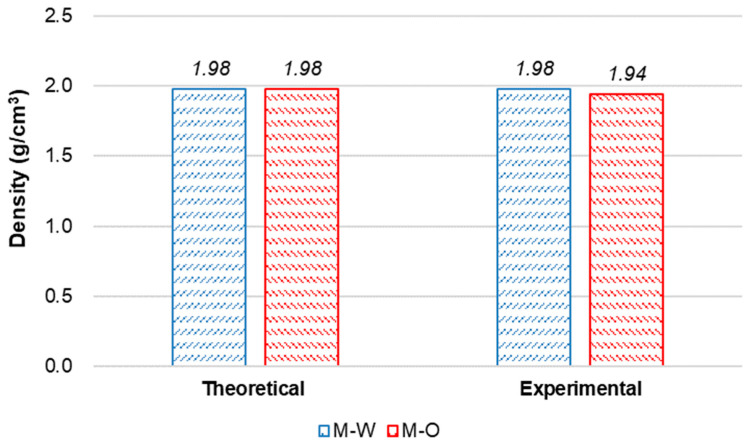
Theoretical and experimental densities of SHCC.

Figure 2 shows theoretical and experimental densities of the M-W and M-O mixtures. Theoretical density values were calculated based on the density of each material and its amount. As can be seen, differences between theoretical and experimental densities of M-W and M-O mixtures were 0% and 2.0%, respectively. This indicates that volume of air trapped in the M-O mixture was slightly higher than that in the M-W mixture, and that the matrix of the M-O mixture might be more porous than that of the M-W mixture.

**Figure 3:**
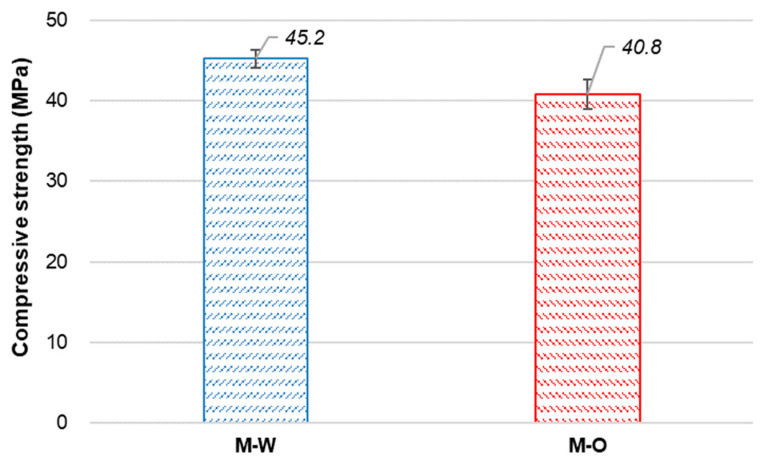
Compressive strength of SHCC.

Figure 3 shows the compressive strength ($f_{c}$) of the M-W and M-O mixtures at 28 days of age. The value of $f_{c}$ of the M-O mixture was 10% lower than that of the M-W mixture, which is likely due to the denser matrix of the M-W mixture (Figure 2). It can be hypothesized that U-C, by promoting further hydration and crystal precipitation to create new material to fill pores existing inside the matrix, enhanced the $f_{c}$ of SHCC more than N-C.

**Figure 4:**
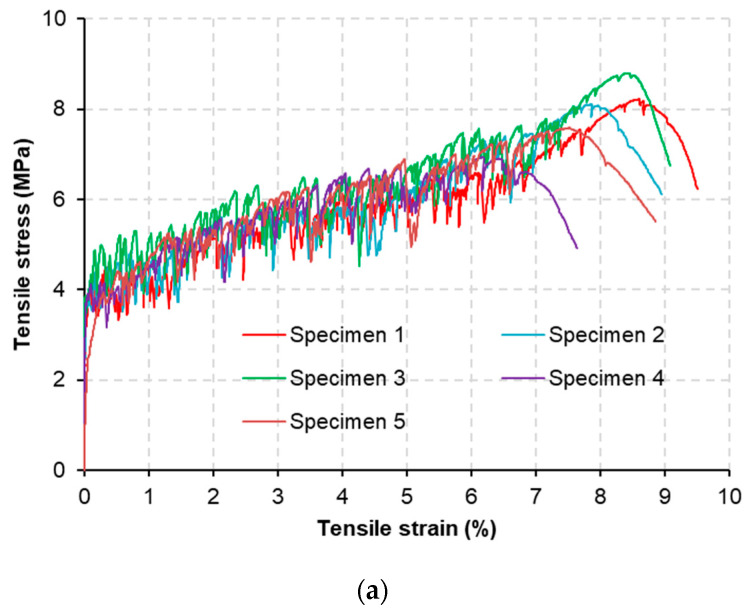

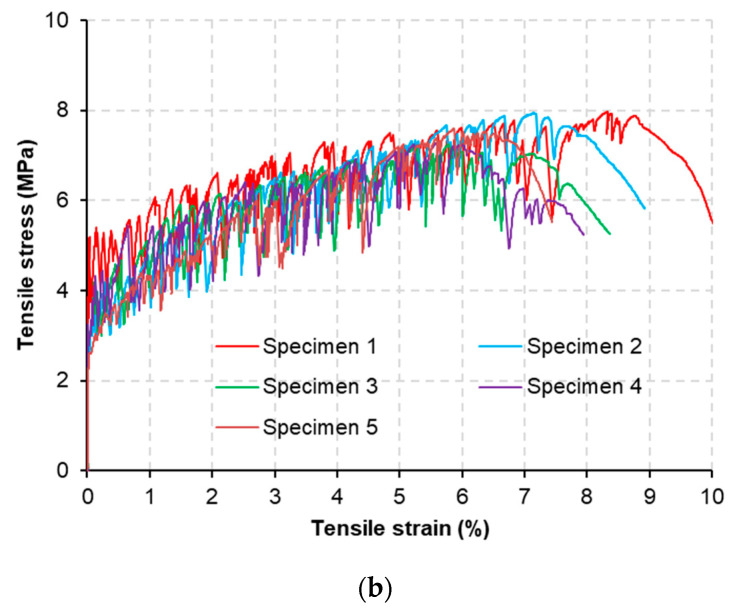
Stress–strain curves of: (**a**) M-W and (**b**) M-O mixtures.

Figure 4 shows the tensile stress–strain curves of the M-W and M-O mixtures at 28 days of age. The two mixtures clearly exhibit strain-hardening responses with many stress fluctuations, indicating the creation of multiple cracks. Both mixtures achieved ultra-ductile behavior in accordance with their tensile strain capacity values of approximately 8%. The tensile properties of the M-W and M-O mixtures, including first cracking strength ($f_{cr}$), ultimate tensile strength ($f_{ts}$), tensile strain capacity ($\varepsilon_{c}$), and toughness ($E_{t}$), are detailed in Table 5. The $f_{ts}$ values of these two mixtures were higher than their own $f_{cr}$ values, indirectly confirming that the strain-hardening criteria had been met. Similar to the trend of compressive strength, values of $\;f_{cr}$ and $f_{ts}$ of the M-O mixture were lower than those of the M-W mixture by 16% and 4%, respectively. In addition, values of $\varepsilon_{c}$ and $E_{t}$ of the M-O mixture were lower than those of the M-W mixture by 3% and 11%, respectively. It seems that U-C plays an essential role in improving the interfacial properties between fiber and matrix, much better than the role that N-C played in SHCC. Overall, the M-O mixture had a tensile performance slightly lower than that of the M-W mixture. The ratio values of $f_{cr}$/$f_{c}$ of the M-W and M-O mixtures were 8.3% and 7.7%, respectively; these values were similar to those of conventional concrete. On the other hand, the two mixtures showed the potential to improve the structural performance because their values of $f_{ts}$/$f_{c}$ were approximately 20% [16].

**Figure 5:**
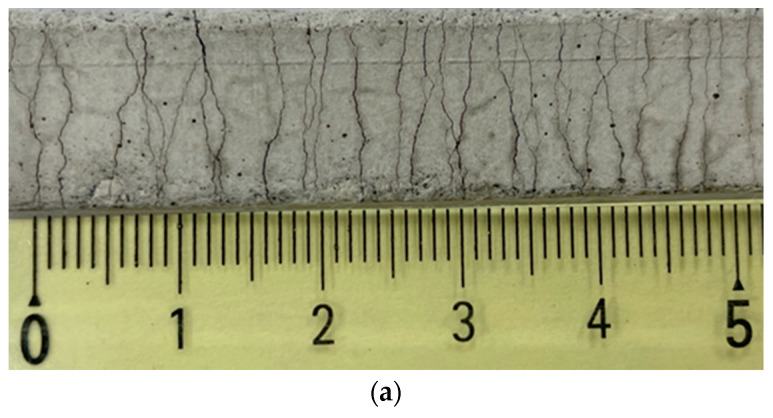

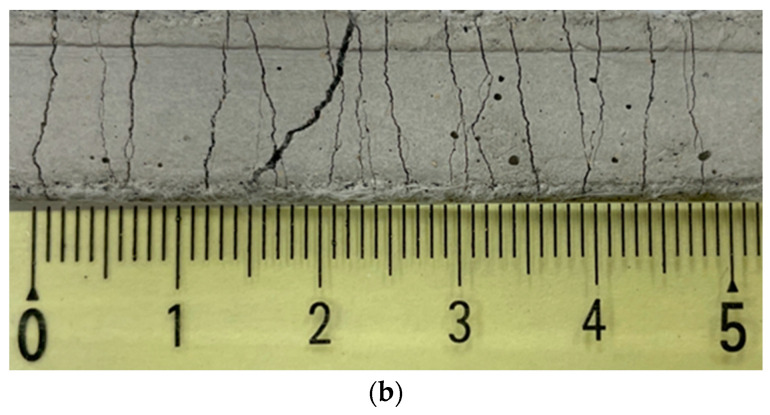
Cracking patterns of: (**a**) M-W and (**b**) M-O mixtures.

Figure 5 shows representative cracking patterns of the M-W and M-O mixtures. It seems that the M-W mixture exhibited a larger number of saturated cracks with tight crack width than the M-O mixture. Detailed information on the cracking patterns, including number of cracks ($\eta_{c}$), average crack width ($\omega_{c}$), and crack spacing ($l_{c}$), is provided in Table 6. The value of $\eta_{c}$ of the M-W mixture was higher than that of the M-O mixture by 61%; however, the values of $\omega_{c}$ and $l_{c}$ of the M-W mixture were 37% and 39% lower than those of the M-O mixture, respectively. Based on ACI 318–1995 [30,31], the crack width of concrete members should be smaller than 0.30 mm to guarantee their use in exterior applications. Accordingly, SHCC has great potential to improve structural performance in accordance with its value of $\omega_{c}$ smaller than 0.15 mm.

### 3.2. Crack Healing Performance

**Figure 6:**
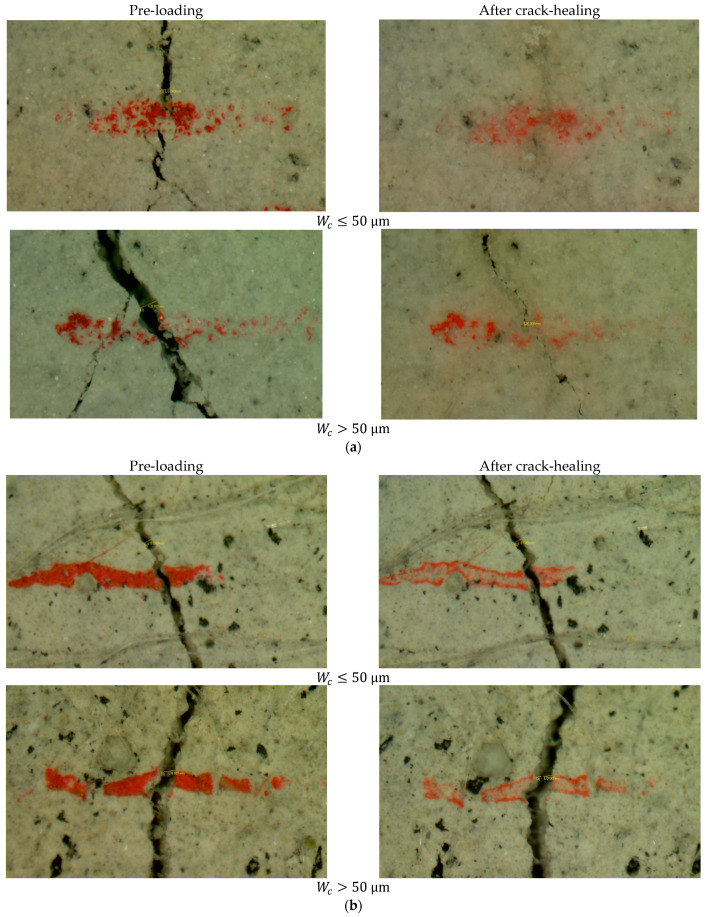
Representative crack healing phenomena of: (**a**) M-W and (**b**) M-O mixtures.

**Figure 7:**
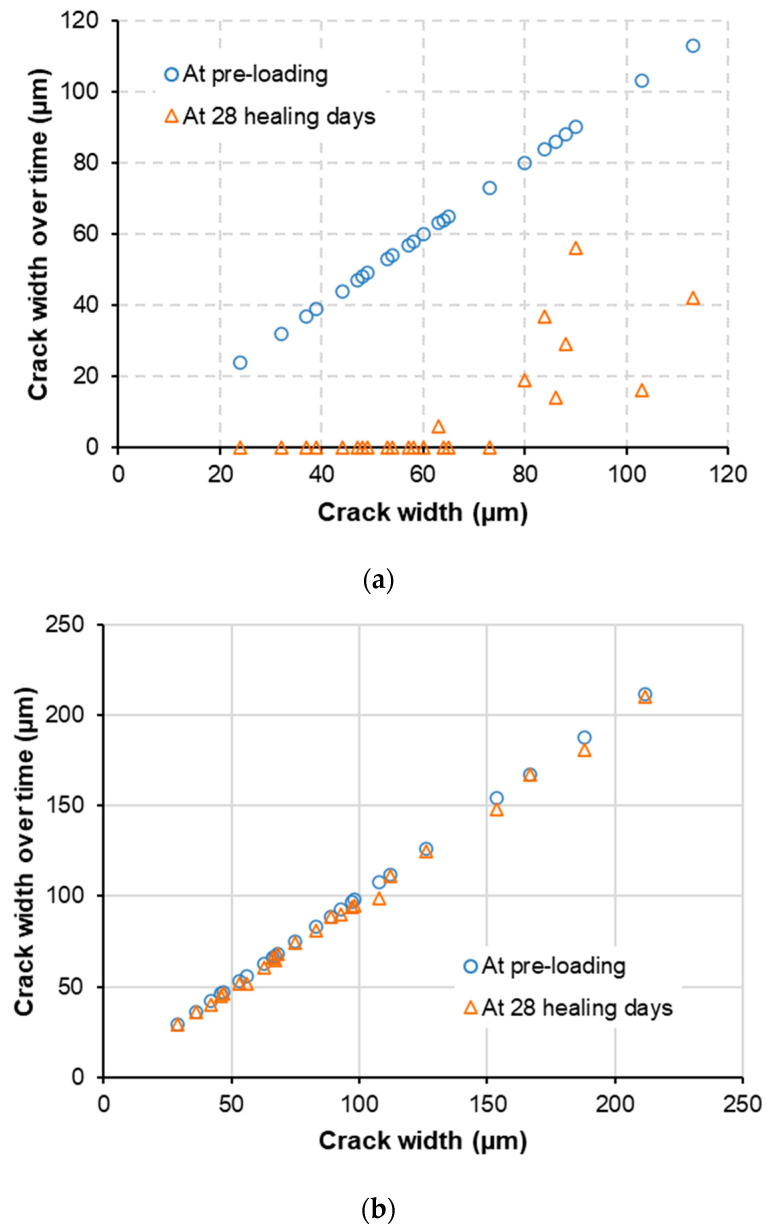
Crack width reduction of: (**a**) M-W and (**b**) M-O mixtures.

Figure 6 shows representative crack healing phenomena of M-W and M-O mixtures. It is clear that the M-W mixture showed excellent healing performance for both small ($W_{c}\leq 50$ μm) and large ($W_{c}>50$ μm) cracks. On the other hand, the M-O mixture had very limited healing performance, even though the materials and mixture proportions of the M-O mixture were identical to those of the M-W mixture. The healing materials sealing the original cracking zones of the M-W mixture seem to be precipitated as crystalline products. Figure 7 shows initial values of $W_{c}$ at pre-cracking and residual values of $W_{c}$ after 28 healing days. The selected initial $W_{c}$ of the M-O mixture ranged from 30 µm to 210 µm, approximately two times higher than the range of the M-W mixture. The reason for this is that the value of $\omega_{c}$ of the M-O mixture was about 1.6 times higher than that of the M-W mixture (Table 6). As shown in Figure 7, crack-healing becomes possible once healed $W_{c}$ becomes smaller than initial $W_{c}$. Generally, the M-W mixture obtained better crack healing capacity than that of the M-O mixture because almost all residual $W_{c}$ values of the M-W mixture were equal to zero in the final healing stage. In contrast, residual $W_{c}$ values of the M-O mixture were nearly unchanged over the healing timeline. The healing threshold of the M-W mixture was approximately 60 µm, the same as the value reported in the literature [8]. To quantitatively evaluate the healing performance, the healing rate ($\gamma_{r}$) was defined by Equation (2).

(2) $$\gamma_{r}=\left(1-\frac{W_{c-h}}{W_{c-i}}\right)\times 100\%$$

where $W_{c-h}$ is the value of $W_{c}$ after 28 healing days and $W_{c-i}$ is the initial value of $W_{c}$ at pre-cracking.

**Figure 8:**
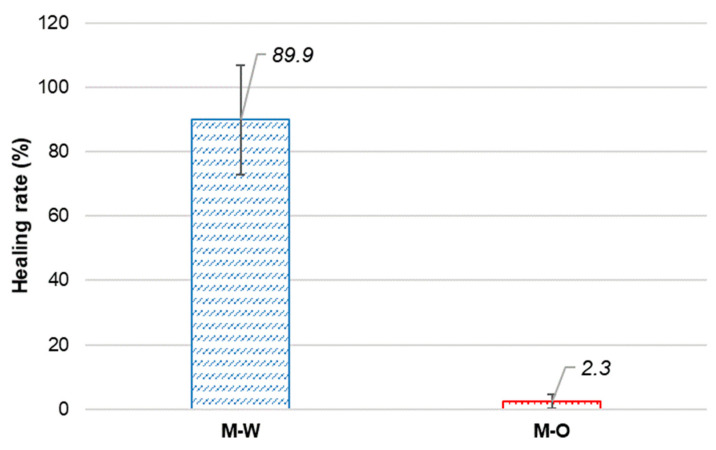
Healing rate of SHCC.

Figure 8 shows the healing rates of the two mixtures. The value of $\gamma_{r}$ of the M-W mixture completely overwhelms that of the M-O mixture, being 38.6 times higher. Accordingly, it can be concluded that the crack healing performance of SHCC investigated in this study is significantly influenced by environmental conditions. As has been reported, water can provoke further hydration of unhydrated cement. Then, new hydration products are deposited within cracking zones, filling them. It was found that N-C, which involves rainwater and sunshine, was not effective at activating crack-healing of the M-O mixture. It is likely that the rainwater absorbed inside the cracking portions of the specimens but did not sufficiently diffuse the chemical components to generate good hydration, or the rainfall may have to be higher to enable full healing for the M-O mixture. Crack-healing SHCC is thus very feasible for use in underwater infrastructure in which the availability of water can facilitate the healing process.

### 3.3. Stiffness Recovery

**Figure 9:**
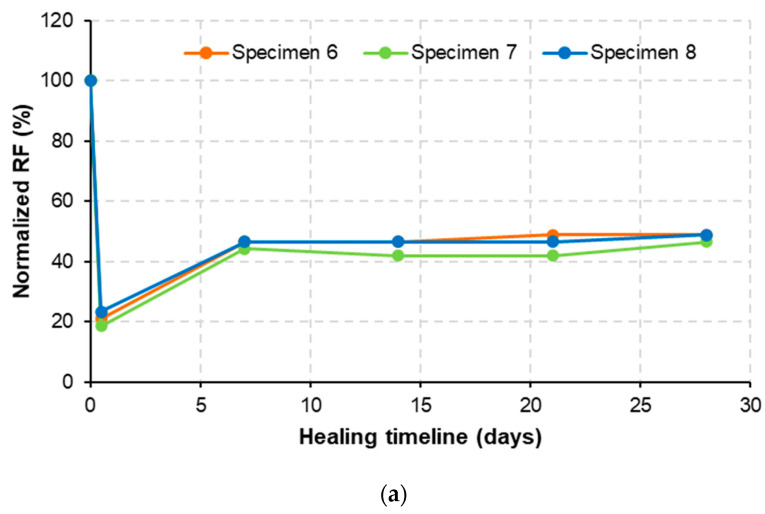

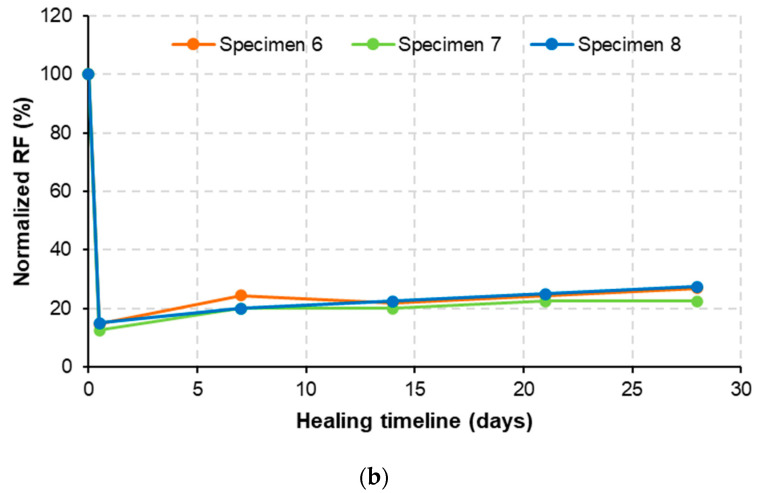
Normalized RF of: (**a**) M-W and (**b**) M-O mixtures.

Figure 9 shows the normalized value of RF of SHCC; normalized RF ($RF_{n}$) was calculated using Equation (3).

(3) $$RF_{n}=\frac{RF_{h}}{RF_{i}}\times 100\%$$

where $RF_{i}$ is RF of the undamaged specimen and $RF_{h}$ is RF at a specific healing timeline.

After pre-cracking, the normalized RF values of the M-W and M-O mixtures were reduced by about one-fifth compared to the original values. This was because pre-cracks broke down the structural integrity of the specimens, significantly lessening wave transmission caused by impact load. Later, normalized RF values of the two mixtures increased gradually over the healing timeline, implying that healing materials were created inside the pre-cracks, partially sealing cracking gaps and improving the stiffness property of composite structures. At 28 healing days, the normalized RF values of the M-W and M-O mixtures were higher than the initial normalized RF values by 27%p and 12%p, respectively. This indicates that both mixtures had crack-healing potential upon stiffness recovery; however, the M-W mixture acquired much better healing properties than the M-O mixture. It is necessary to mention that the M-O mixture showed a relative stiffness recovery but low crack sealing capacity (Section 3.2). It is likely that the healing materials formed on the cracking surfaces of the M-O mixture were washed out by the rainfall.

### 3.4. Tensile Properties at Post-Healing

**Figure 10:**
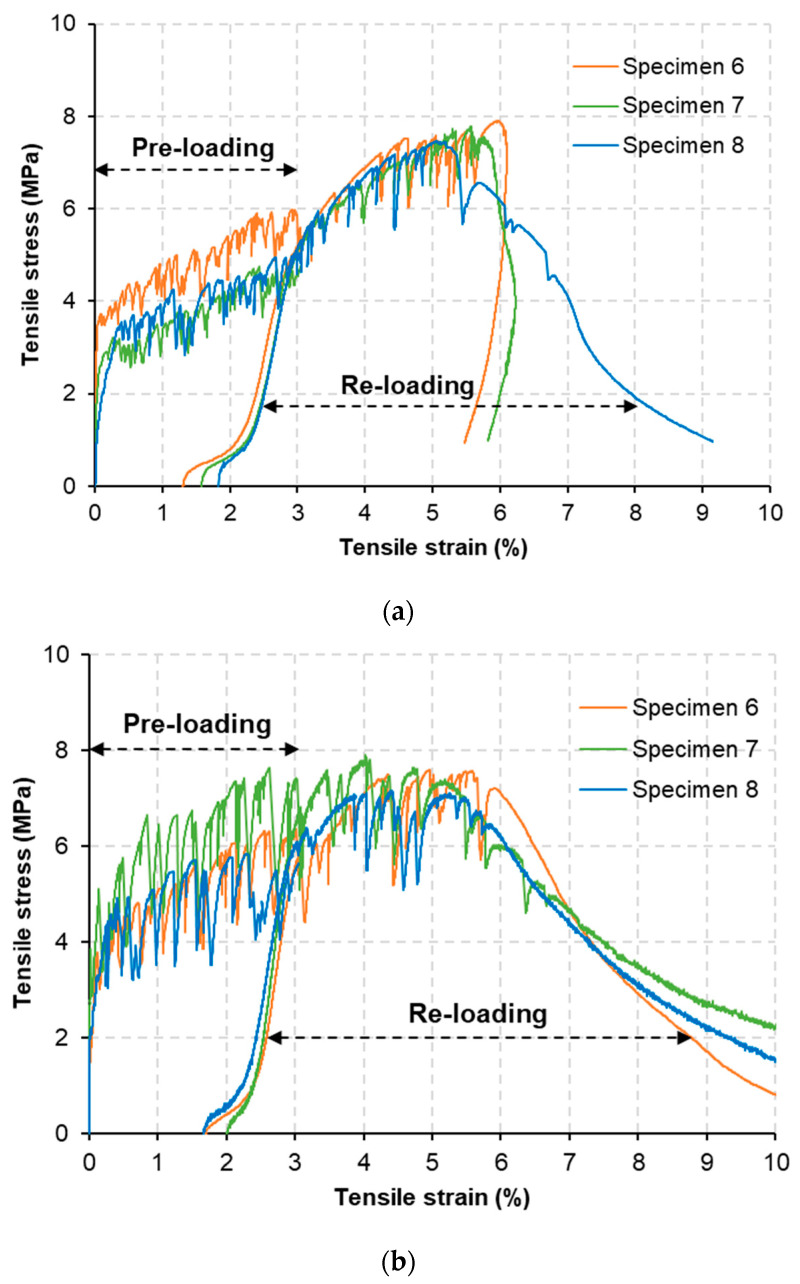
Tensile stress–strain curves at post-healing of: (**a**) M-W and (**b**) M-O mixtures.

**Figure 11:**
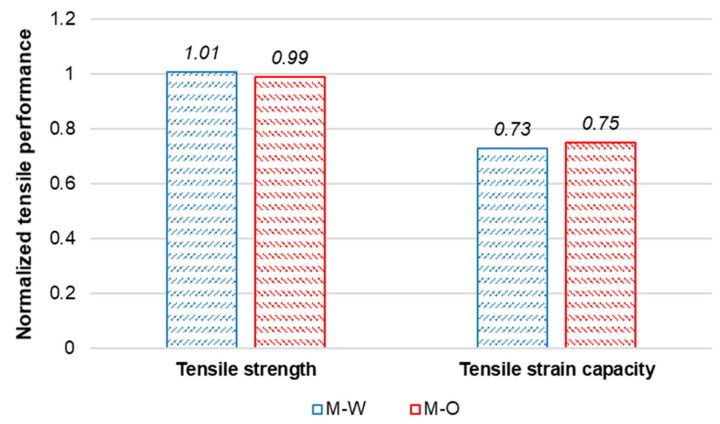
Normalized tensile properties after crack-healing.

Figure 10 shows tensile stress–strain curves describing pre-loading and re-loading phases of the M-W and M-O mixtures. The reloading curves were drawn according to the cyclic loading condition to eliminate residual tensile strain of pre-cracks when they re-opened. The specimens of the two mixtures showed strain-hardening behavior; however, their tensile ductility decreased compared to those of specimens tested at 28 days of age. Table 7 lists the tensile strength ($f_{ts-h}$) and tensile strain capacity ($\varepsilon_{c-h}$) values at post-healing of the M-W and M-O mixtures, determined from the re-loading curves. Figure 11 shows normalized tensile performance at post-healing compared to that at 28 days of age (Table 5). Although the two mixtures showed quite different levels of crack-healing performance, they showed similar normalized tensile performance at re-loading after 28 healing days. For two mixtures, their $f_{ts-h}$ was almost unchanged compared to that of $f_{ts}$ while their $\varepsilon_{c-h}$ was reduced a quarter compared to that of $\varepsilon_{c}$. It is likely that the healing materials attached along the fibers bridging existing cracks might induce an increase in the interfacial bonding strength, which resulted in decreased crack width. Increased cracking strength might also decrease the number of cracks.

## 4. Conclusions

The study experimentally investigated the effects of natural and underwater conditions on the mechanical properties and crack-healing capacity of hybrid fiber-reinforced SHCCs. Based on the experimental results, the following conclusions can be drawn:

1. At 28 days of age, the compressive strength of the M-O mixture was 10% lower than that of the M-W mixture, which indicates that underwater conditions were better than natural conditions outdoors at improving the compressive strength of SHCC.
2. Under static tension load, M-W and M-O mixtures showed ultra-ductile behavior, with tensile strain capacity of approximately 8% at 28 days of age. It was observed that the tensile strength and tensile strain capacity of the M-O mixture were lower than those of the M-W mixture by 4% and 3%, respectively.
3. Over 28 healing days, the M-W mixture showed complete crack healing phenomena, with healing threshold of crack width of approximately 60 µm, while the M-O mixture had very limited crack healing capacity. Both M-W and M-O mixtures showed potential for stiffness recovery; however, the stiffness recovery of the M-O mixture was 15%p lower than that of the M-W mixture. Accordingly, the natural condition is not sufficient for high crack-healing of SHCC, while the underwater condition plays an essential role in facilitating excellent crack-healing performance.
4. The M-W and M-O mixtures showed clear strain-hardening responses in the re-loading stage. After crack-healing, the tensile strengths of the two mixtures were unchanged; however, their tensile strain capacities were relatively reduced compared to those at pre-healing.

## Figures and Tables

**Table 1:** Chemical composition of cement and GGBS.

Materials	SiO_2_	Al_2_O_3_	Fe_2_O_3_	CaO	MgO	SO_3_	TiO_2_	K_2_O	Na_2_O	Other
Cement	8.20	2.70	5.02	66.40	0.60	2.80	0.21	-	9.10	4.97
GGBS	34.95	13.58	0.53	42.88	3.58	2.52	0.63	0.61	0.26	0.46

**Table 2:** Physical properties of PE and PVA fibers.

Fiber	Length (mm)	Diameter (µm)	Tensile strength (MPa)	Density (g/cm^3^)	Elastic modulus (GPa)
PE	12	16	2700	0.97	88
PVA	8	40	1560	1.30	41

**Table 3:** Mixture proportions and curing methods.

Mixture	Binder		Water	CR	SS	EA	SP	VMA	DF	Fiber		Curing
	Cement	GGBS								PE	PVA	
M-W	0.5	0.5	0.4	0.1	0.5	0.05	0.002	0.001	0.001	1.25	0.1	U-C
M-O	0.5	0.5	0.4	0.1	0.5	0.05	0.002	0.001	0.001	1.25	0.1	N-C

**Table 4:** Information on natural environment.

Age (Days)	Days of Rain	Precipitation (mm)		Average Temperature (°C)	Average Relative Humidity (°C)
		Total	1-Day (Avg.)		
3~28	8	143	5.5	28.5	75.2
29~56	18	274	9.8	25.5	84.3

**Table 5:** Tensile properties of SHCC.

Mixture	$f_{cr}\;(\mathrm{MPa})$	$f_{ts}\;(\mathrm{MPa})$	$\varepsilon_{c}\;(\%)$	$E_{t}\;(\mathrm{MPa}\;m/m)$
M-W	3.74 ± 0.19	7.93 ± 0.62	8.00 ± 0.69	0.46
M-O	3.13 ± 0.43	7.60 ± 0.31	7.74 ± 0.63	0.41

**Table 6:** Detailed information on cracking patterns of SHCC.

Mixture	$\eta_{c}$	$\omega_{c}\;(µm)$	$l_{c}\;(\mathrm{mm})$
M-W	67.1 ± 6.0	95.6 ± 5.6	1.20 ± 0.11
M-O	41.6 ± 6.6	151.2 ± 17.6	1.96 ± 0.26

**Table 7:** Tensile strength and tensile strain capacity at post-healing.

Mixture	$f_{ts-h}\;(\mathrm{MPa})$	$\varepsilon_{c-h}\;(\%)$
M-W	7.72 ± 0.18	5.85 ± 0.13
M-O	7.54 ± 0.30	5.79 ± 0.05

## Data Availability

Not applicable.
